# Revisiting the Dependence of Poisson’s Ratio on Liquid Fragility and Atomic Packing Density in Oxide Glasses

**DOI:** 10.3390/ma12152439

**Published:** 2019-07-31

**Authors:** Martin B. Østergaard, Søren R. Hansen, Kacper Januchta, Theany To, Sylwester J. Rzoska, Michal Bockowski, Mathieu Bauchy, Morten M. Smedskjaer

**Affiliations:** 1 Department of Chemistry and Bioscience, Aalborg University, 9220 Aalborg East, Denmark; 2 Institute of High-Pressure Physics, Polish Academy of Sciences, 01-142 Warsaw, Poland; 3 Department of Civil and Environmental Engineering, University of California, Los Angeles, CA 90095, USA

**Keywords:** oxide glasses, poisson’s ratio, liquid fragility, atomic packing density

## Abstract

Poisson’s ratio (*ν*) defines a material’s propensity to laterally expand upon compression, or laterally shrink upon tension for non-auxetic materials. This fundamental metric has traditionally, in some fields, been assumed to be a material-independent constant, but it is clear that it varies with composition across glasses, ceramics, metals, and polymers. The intrinsically elastic metric has also been suggested to control a range of properties, even beyond the linear-elastic regime. Notably, metallic glasses show a striking brittle-to-ductile (BTD) transition for ν-values above ~0.32. The BTD transition has also been suggested to be valid for oxide glasses, but, unfortunately, direct prediction of Poisson’s ratio from chemical composition remains challenging. With the long-term goal to discover such high-ν oxide glasses, we here revisit whether previously proposed relationships between Poisson’s ratio and liquid fragility (*m*) and atomic packing density (*C*_g_) hold for oxide glasses, since this would enable *m* and *C*_g_ to be used as surrogates for *ν*. To do so, we have performed an extensive literature review and synthesized new oxide glasses within the zinc borate and aluminoborate families that are found to exhibit high Poisson’s ratio values up to ~0.34. We are not able to unequivocally confirm the universality of the Novikov-Sokolov correlation between *ν* and *m* and that between *ν* and *C*_g_ for oxide glass-formers, nor for the organic, ionic, chalcogenide, halogenide, or metallic glasses. Despite significant scatter, we do, however, observe an overall increase in *ν* with increasing *m* and *C*_g_, but it is clear that additional structural details besides *m* or *C*_g_ are needed to predict and understand the composition dependence of Poisson’s ratio. Finally, we also infer from literature data that, in addition to high *ν*, high Young’s modulus is also needed to obtain glasses with high fracture toughness.

## 1. Introduction

Poisson’s ratio (*ν*) is the negative ratio of the transverse strain to the longitudinal strain of a material under uniaxial stress in the elastic regime. It relates to the shear modulus (*G*) and bulk modulus (*B*), as

(1) $$\nu =\frac{3B-2G}{6B+2G}$$

For isotropic materials in three dimensions [1], this limits *ν* to be within −1 and 0.5, as the values of *G* and *B* are always positive. Different material families and compositions exhibit pronounced diversity in their elastic properties and thus Poisson’s ratio. Materials with *ν* ~ 0.5 are highly incompressible and tend to deform through shape change, while materials with *ν* ~ 0 are highly compressible. So-called auxetic materials, with negative values of *ν*, swell under tension [2,3,4]. At *ν* ~ 0.2, a transition between two different types of stress patterns in frozen-in solid has been reported, namely shear and uniform deformation [5]. Various macroscopic properties have been linked to Poisson’s ratio [6], including some outside the elastic regime, such as densification [7], connectivity [8], and ductility [9].

**Figure 1:**
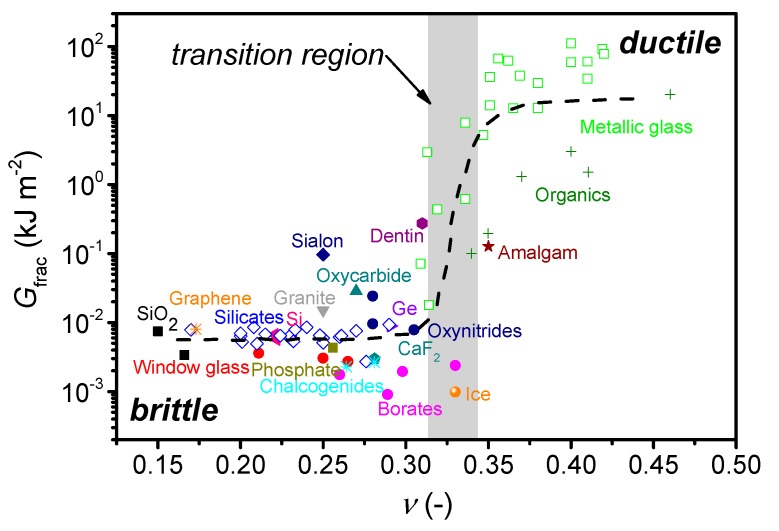
Dependence of fracture energy (*G*_frac_) on Poisson’s ratio (*ν*) for a range of materials, showing a brittle-to-ductile transition in the range of *ν* from 0.30 to 0.33. The figure is reproduced with the data from Lewandowski et al. [9] and Tian et al. [17]. We also extend it with new *G*_frac_ data for silicate glasses [10,18,19,20,21], borate, chalcogenide, and metallic glasses [10,20], and graphene [22,23] obtained by single-edge pre-crack beam (SEPB), chevron notch (CN), single edge notch beam (SENB), indentation fracture (IF), or tensile testing methods. The error of *ν* and *G*_frac_ is estimated to be 0.01 and 15%, respectively. The dashed line is a guide for the eye.

Oxide glasses exhibit interesting properties such as transparency, high hardness, high chemical durability (in many cases), and low-cost of raw materials. The brittleness of oxide glasses has been a major hindrance for their use in various engineering and functional applications [10]. As the crack tip formation and growth mechanisms are not well understood, it is challenging to design ductile oxide glasses. Post-processing approaches such as chemical strengthening [11] are thus currently used to improve the mechanical performance. However, molecular dynamics (MD) simulations suggest that silicate glasses can exhibit some nanoscale ductility [12,13], and it is also possible for silica glass to feature ductility induced by electron-beam irradiation [14]. Interestingly, as shown for metals [15] and metallic glasses [9], high *G*/*B* ratio (and thus low Poisson’s ratio) favors brittleness. In other words, a correlation between fracture energy (*G*_frac_), i.e., energy required to create two new fracture surfaces, and Poisson’s ratio has been observed, which also manifests itself by a brittle-to-ductile (BTD) transition around *ν*_BTD_ = 0.32 not only for metallic glasses but various non-crystalline solids (Figure 1) [6,9,16]. 

The problem for oxide glasses is the fact that they mostly exhibit *ν* < 0.30, with only few oxide glasses reported with *ν* > 0.34 [24,25]. As such, the existence of a BTD transition for oxide glasses needs additional verification. However, recent MD simulations on permanently densified SiO_2_ glasses have confirmed the existence of a BTD transition, although the value of *ν*_BTD_ was found to depend on the average coordination number [26]. Moreover, a recent study has explained the empirical BTD transition based on microscopic dynamical properties [16], building on the observation that ductility is closely related to the secondary *β*-relaxation [27,28], while Poisson’s ratio is proposed to be related to the effective Debye-Waller factor. The study suggests that ductile materials can withstand deformation at higher rates because they exhibit faster *β*-relaxation [16]. 

In an attempt to overcome the brittleness of oxide glasses, it is thus of great interest to discover high-*ν* oxide glasses (*ν* > 0.32). Unfortunately, there are presently no composition-dependent models available for predicting *ν*, and thus, inefficient Edisonian trial-and-error composition design is currently utilized [29]. It is therefore of interest to find predictable surrogates for Poisson’s ratio. Most notably, liquid fragility (*m*) has been proposed to be positively correlated with the ratio of bulk and shear moduli (and thus Poisson’s ratio) for a broad range of glassy systems covering covalent and hydrogen-bonded, van der Waals and ionic glasses, i.e., a range of organic molecules, oxide, halogenide, and chalcogenide glasses [30,31]. Angell’s liquid fragility is defined as the slope of the base-10 logarithm of viscosity versus *T*_g_-scaled inverse temperature curve at *T*_g_, where *T*_g_ is the glass transition temperature (*m* = *d* log (*η*)/*d* log (*T*_g_/*T*) at *T*_g_) [32]. This is the fragility index used in this work, although we note that other definitions of fragility exist [33]. The proposed *m*-*ν* relation is of interest, since tools such as topological constraint theory [34,35] and coarse-graining (related to structural connectivity) [36,37] can be used to predict *m*. Since the original study by Novikov and Sokolov in 2004 [30], a similar *m*-*ν* dependence has been found for metallic glasses, although the change in fragility with modulus ratio varies for different systems [38,39,40]. It has been noted that the correlation is only observed within a narrow range of *m* due to the limited amount of data on bulk metallic glasses [40]. The proposed linear relationship between *m* and the bulk-to-shear modulus has been seriously questioned by Yannopoulos and Johari [41], who have argued that some data points were erroneously plotted, showing that no general correlation for neither organic, inorganic, nor metallic glasses exists when including more data. The lack of correlation between *m* and elastic properties has then been suggested to be due to the strong sensitivity of *ν* to temperatures above *T*_g_, as strong melts (low *m*) exhibit Poisson’s ratio that is almost constant before and after the glass transition, while fragile melts (high *m*) show a significant change in Poisson’s ratio above *T*_g_ [8]. There have been studies supporting the *m*-*ν* correlation. For example, building on a proposed relation between Poisson’s ratio and packing density (see below) Duval et al. [42] argue that the relation between *m* and *ν* is due to the structural fluctuations being breathing-like (with change of volume) in strong liquids and shear-like (without change of volume) in fragile liquids. Greaves et al. [6] have also argued that the correlation between *m* and *ν* depend on the glass system, showing linear correlations for binary alkali silicates and metallic glasses, but with different slopes.

Besides liquid fragility, Poisson’s ratio has been suggested to be positively correlated with the atomic packing density (*C*_g_) [6,8,43], which is defined as the ratio between the volume occupied by the ions and the corresponding effective volume of glass. *C*_g_ could potentially be a good surrogate for Poisson’s ratio, since the compactness of the sample affects the vibrational modes [42] and materials with a high *C*_g_ should exhibit relatively strong interatomic interactions [16]. Based on experimental data, an empirical relation between *ν* and *C*_g_ has been proposed (*ν* = 0.5–1/7.2*C*_g_) [43], but it has been found to overestimate the Poisson’s ratio for borate and phosphate glasses and underestimate it for germanate and aluminate glasses [8]. A reason for an overestimated Poisson’s ratio of borate glasses might be the low average coordination number as explained for the prediction of Young’s modulus [44].

The purpose of this work is to revisit the validity and universality of the proposed *m*-*ν* and *C_g_*-*ν* correlations. This is done to determine whether prediction of liquid fragility or atomic packing density can be used to guide the discovery of oxide glasses with high Poisson’s ratio (*ν* > 0.32), which are expected to be ductile following the relation in Figure 1. To do so, we perform an extensive literature review to obtain liquid fragility, density, and Poisson’s ratio data for various glass systems. Since experimental data on oxide glasses with *ν* > 0.30 are scarce, we also synthesize a total of 20 new oxide glasses, particularly aluminoborate and zinc borate glasses as these have been found to have relatively high Poisson’s ratio [45,46]. To further expand the dataset, we also determine the missing property (e.g., *m* if only *C_g_* and *ν* are known) from previously synthesized glasses in our laboratory [18,46,47,48,49]. Moreover, we subject selected oxide glasses to high-temperature densification to induce a higher *C*_g_ value in bulk samples and then probe whether it correlates with an expected increase in *ν.* Finally, we also discuss the implications of the findings for designing tough oxide glasses. 

## 2. Experimental

### 2.1. Sample Preparation

Oxide glasses were prepared by the traditional melt-quenching technique using reagent grade chemicals (see Table 1). Three families of glasses were synthesized, namely Zn-borates, aluminoborates, and Ca-Zr-silicates. Zn-borates were prepared from H_3_BO_3_ (Hoenywell, North Caroline, USA) and ZnO (VWR, Leuven, Belgium), and doped with La_2_O_3_ (Sigma-Aldrich, Steinheim, Germany), Ta_2_O_5_ (Sigma-Aldrich, Steinheim, Germany), and/or GeO_2_ (Alfa Aesar, Massachusetts, USA). Aluminoborates were prepared from H_3_BO_3_ and Al_2_O_3_ (Sigma-Aldrich, Steinheim, Germany) with additions of BaCO_3_ (ChemPUR, Karlsruhe, Germany), MgCO_3_ (Acros Organics, New Jersey, USA), CaCO_3_ (Sigma-Aldrich, Steinheim, Germany), Li_2_CO_3_ (Merck, Darmstadt, Germany), Cs_2_CO_3_ (Sigma-Aldrich, Steinheim, Germany), Ga_2_O_3_ (Sigma-Aldrich, Steinheim, Germany), and/or Ta_2_O_5_ (Sigma-Aldrich, Steinheim, Germany). Ca-Zr-silicates were produced from SiO_2_ (Merck, Darmstadt, Germany), ZrO_2_ (Hoenywell, North Caroline, USA), and CaCO_3_. All glasses were post-annealed for 30 min at around *T*_g_ (*T*_g_ ± 5 °C) (*T*_g_ is determined by differential scanning calorimetry), prior to density and Poisson’s ratio characterization to ensure similar thermal history.

Some of the synthesized glasses (sample size approx. 13 × 13 × 2.5 mm^3^) were then subjected to isostatic compression at their respective ambient pressure *T*_g_ value in a nitrogen gas pressure chamber containing a multizone cylindrical furnace [50]. The applied pressure was 1 GPa and the compression time was 30 min, which is needed for obtaining a fully densified structure [51]. After the treatment the samples were first cooled to room temperature, then relaxed to ambient pressure at room temperature (but the glasses remain partially densified).

### 2.2. Characterization

We determine the values of *m*, *C_g_*, and *ν* for both the newly-synthesized glasses and those missing from previous studies [18,46,47,48,49], as shown in Table 1. First, the densities (*ρ*) of the glasses were determined using the Archimedes principle with ethanol as the immersion medium. The measured density and chemical composition were used to calculate the molar volume (*V*_m_) and in turn atomic packing factor (*C*_g_) using Equations (2) and (3), respectively.

(2) $$V_{m}=\frac{1}{\rho }\sum_{i}x_{i}M_{i}$$

(3) $$C_{g}=\frac{1}{V_{m}}\sum_{i}x_{i}V_{i}.$$

Here *x*_i_, *M*_i_, and *V*_i_ are the mole fraction, molar mass, and ionic volume (or metallic radii), respectively, of each compound. Structural assumptions regarding valence and coordination number of each cation are described in detail in the Supporting Information, while the anionic oxygen, nitrogen, and fluorine radii were assumed to be 1.35, 1.46, and 1.285 Å, respectively, as reported by Shannon [53]. The packing density of metallic glasses was calculated by using the metallic radii of the pure metal [54], as suggested by Rouxel [8].

Given the small sample size for many of the studied glasses (due to their poor glass-forming ability), we did not determine the liquid fragility (*m*) using direct viscosity measurements. Instead we determined it using differential scanning calorimetry (DSC) on a STA 449F1 instrument (Netzsch, Selb, Germany). Small disc-shaped specimens of 40–60 mg and Ø ~ 4 mm were prepared for measurements. DSC upscans were performed in Pt crucibles and argon atmosphere (50 mL/h) at different heating rates subsequent to cooling the glasses from well above the glass transition at the same rate. The heating/cooling rates were 5, 10, 20, and 30 °C/min. The fragilities were corrected for a systematic error using Equation (4), as described in Zheng et al. [55].

(4) $$m=1.289\left(m_{\mathrm{DSC}}-m_{0}\right)+m_{0}$$

Here, *m*, *m*_DSC_, and *m*_0_ are the liquid fragility determined from viscosity, the liquid fragility determined from DSC, and the fragility of a perfectly strong glass that equals 14.97, respectively.

Samples were ground using SiC paper to obtain coplanar surfaces. The longitudinal and transverse wave velocities (*V*_L_ and *V*_T_, respectively) were measured by an ultrasonic thickness gauge (38DL Plus; Olympus, Tokyo, Japan) using the pulse-echo method with 20 MHz delay line. The thickness of the samples were measured with a digital micrometer (Mitutoyo, Kawasaki, Japan) with a precision of 0.01 mm. Poisson’s ratio (*ν*) was calculated from *V*_L_ and *V*_T_, following Equation (5). For literature studies, which did not report the value of *ν*, it was calculated either from wave velocities using Equation (5), or from the other elastic moduli based on the isotropic nature of the oxide glasses (see, e.g., Equation (1)).

(5) $$\nu =\frac{V_{L}^{2}-2V_{T}^{2}}{2\left(V_{L}^{2}-V_{T}^{2}\right)}$$

## 3. Results and Discussion

### 3.1. Studied Compositions

The compositions of the oxide glasses synthesized and/or characterized in this study are given in Table 1, along with the values of *C*_g_, *T*_g_, *m*, and *ν*. The glasses are made of various network formers, covering silicates, borates, aluminoborates, and aluminoborosilicates with various network-modifying oxides. The glasses also exhibit a wide range of Poisson’s ratio values (approximately from 0.21 to 0.34), and some glasses thus exhibit *ν* > *ν*_BTD_. However, it is outside the scope of the present study to determine the fracture toughness of these glass samples, which, in most cases, are too small in size to be tested via self-consistent fracture toughness methods [10]. The liquid fragility values range from 22 to 60, while the atomic packing density ranges from 0.48 to 0.61. The zinc borate glasses generally feature the highest values of *C*_g_ and *ν*. 

We have identified literature data of Poisson’s ratio using the SciGlass database, in addition to searching traditional glass journals using keywords such as “Poisson’s ratio”, “mechanical properties”, and “elastic properties”. Furthermore, we added the keyword “fragility” to obtain liquid fragility data on similar glass systems. In order to obtain *C*_g_ data, we have identified studies reporting density, molar volume, or *C*_g_ values.

### 3.2. Poisson’s Ratio vs. Packing Density

**Figure 2:**
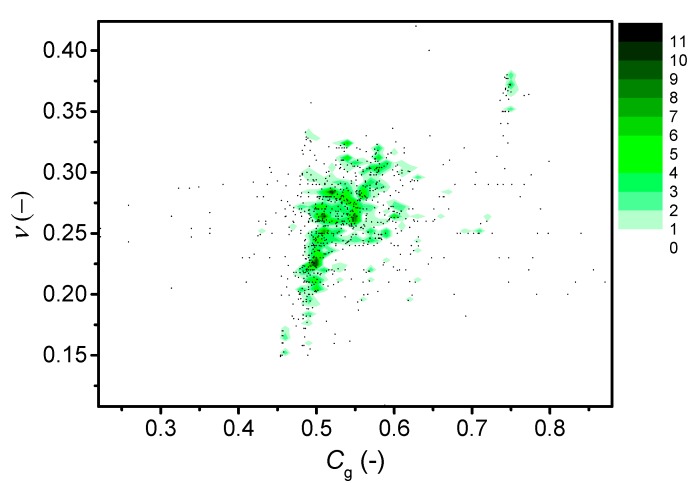
Dependence of Poisson’s ratio (*ν*) on atomic packing density (*C*_g_) for various glass systems, including those from Table 1. The scale represents the multiplicity of data points. References for literature data are given in the text. *C*_g_ is calculated according to Equation (3), building on the structural assumptions described in the Supporting Information. The errors associated with *ν* and *C*_g_ are 0.01 and 0.002, respectively. *R*^2^ value for a sigmoidal fit to the data is 0.162.

Figure 2 shows the dependence of Poisson’s ratio on atomic packing density for various glass systems, covering both the present results and literature data. Due to the large number of points in the center of the plot, Figure 2 shows the density of *C*_g_ vs. *ν* data points for the various glass families, which can be identified in Figure S1 in the Supporting Information. Data for the following glass types are included: metallic [9,20,56,57,58,59], oxynitrides [8,20,60,61], pure oxides [8,20,62,63,64,65,66,67,68,69,70,71,72,73], alkali silicates [8,18,24,63,65,72,74,75], alkali borates [18,64,67,68,76,77,78], alkaline earth silicates [8,79], alkali-alkaline earth borates [80], alkali-alkaline earth silicates (including Pb, Ti, and Fe) [8,20,62,65,67,73,81,82,83,84,85], phosphosilicates [82], germanosilicates [86], aluminosilicates [8,18,60,61,69,70,87,88,89,90,91,92,93,94,95], zinc borates [20,45,76,96], lead borates [20,45,65,96,97,98], aluminoborates [18,46,87,99], germanates [87,100], aluminoborosilicates [18,20,101], borosilicates [18,20,62,67,71,101], borates containing bismuth or tellurium [97,102,103,104,105], halogenides (flourides and oxyflourides) [62,106,107,108,109], vanadates [110,111], tellurites [66,112,113,114,115,116,117,118,119,120,121,122,123,124], phosphates [20,66,68,106,125,126,127,128,129,130,131], rare earth aluminates [8], and oxycarbides [8,20]. In the Supporting Information, we discuss the assumptions (based on structural data) used to calculate *C*_g_ with information from Refs. [18,46,49,53,66,68,76,81,86,87,90,96,97,99,110,111,122,123,125,126,128,130,131,132,133,134,135,136,137,138,139,140,141,142,143,144,145,146,147,148].

We do not observe a strong correlation between *C*_g_ and *ν* (Figure 2), although an overall positive correlation might be apparent, in agreement with the earlier work of Rouxel [8]. However, we note that the present glasses in Table 1 as well as those from literature show a broad range of *ν* values for the same *C*_g_ value, e.g., around *C*_g_ ~ 0.48 (Figure 2). The majority of the data cluster in the center of the diagram, showing an approximate sigmoidal-like trend with a transition of *ν* at *C*_g_ = 0.5. That is, within a limited range of *C*_g_, *ν* increases from around 0.18 to 0.28 for a majority of the glasses, followed by a smaller increase towards *ν* = 0.40 for metallic glasses with *C*_g_ ~ 0.75. As seen in Figure S1 in the Supporting Information, the Makishima-Mackenzie model [43] does not describe the *C*_g_ vs. *ν* trend as well as the sigmoidal-like trend described by Rouxel [8]. Finally, we should note that the majority of studies included in Figure 2 are on silicate and borate glasses, which exhibit similar *C*_g_ values. This is the origin of the clustering of data around *C*_g_ = 0.5 in the plot. On the other hand, many of the data points for phosphate (*ν* = 0.25–0.3) and tellurite (*ν* = 0.2–0.25) glasses are different, with relatively high *C*_g_ (>0.5) and low *C*_g_ (<0.5) values, respectively.

**Figure 3:**
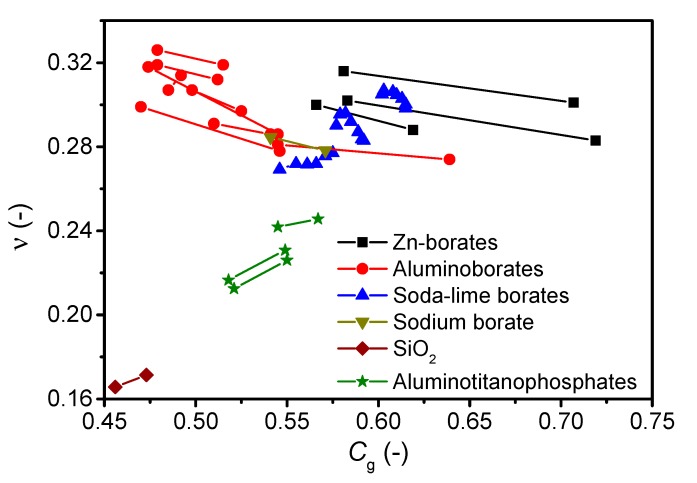
Effect of high-temperature densification, as quantified by the increase in atomic packing factor (*C*_g_), on the Poisson’s ratio (*ν*) of selected glasses: zinc borates (this study), aluminoborates (this study and Ref. [46]), soda-lime borates (Ref. [80]), sodium borate (Ref. [68]), SiO_2_ (Ref. [68]), and aluminotitanophosphates (Ref. [68]). The errors associated with *ν* and *C*_g_ are 0.01 and 0.002, respectively.

In addition to composition variation, the properties of bulk glasses can also change permanently due to post-treatment, such as isostatic high-temperature densification [51,149], which always leads to an increase in *C*_g_ (note that the change in *C*_g_ is measured ex situ under ambient conditions, after the glass is fully decompressed). As shown in Figure 3, the pressure-induced increase in *C*_g_ does not systematically result in an increase in *ν*, casting doubt on the universality of the proposed *C_g_*-*ν* correlation. Zinc borate, aluminoborate, and sodium borate glasses feature a decrease in *ν*, while SiO_2_ and aluminotitanophosphate glasses feature an increase in *ν* upon isostatic compression at 1 GPa around *T*_g_. The soda-lime borate glasses show a complex behavior with a monotonic increase in *ν* and *C*_g_ with increasing pressure (0–0.57 GPa) for low total modifier content (15 mol%), while *ν* first increases for pressures up to 0.2 GPa but then decreases at higher pressures for glasses with higher total modifier content (25 and 35 mol%). The lack of decrease in *ν* with increasing *C*_g_ could be due to the interplay of changing coordination numbers, bond lengths, and bond angles. Densification usually causes two phenomena (i) an increase in *C*_g_, e.g., due to decreased modifier-oxygen bond lengths [150], which typically leads to an increase in Poisson’s ratio, and (ii) an increase in the network connectivity, e.g., through increasing coordination number of network formers, which typically decreases the Poisson’s ratio [8]. These two competitive effects make it difficult to understand the effect of pressure (densification) on the Poisson’s ratio. Finally, we note that a general problem with the calculation of *C*_g_ is the various assumptions needed when insufficient structural data are available [8]. 

### 3.3. Poisson’s Ratio vs. Liquid Fragility

**Figure 4:**
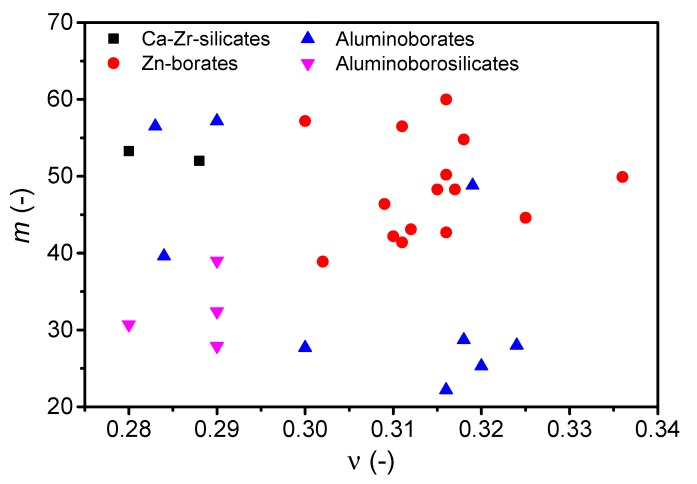
Liquid fragility (*m*) for selected oxide glass-forming systems from this study (Table 1) plotted as a function of Poisson’s ratio (*ν*). No apparent correlation between *m* and *ν* is observed. The errors associated with *m* and *ν* are 1 and 0.01, respectively. *R*^2^ value for a linear fit to the data is 0.034.

**Figure 5:**
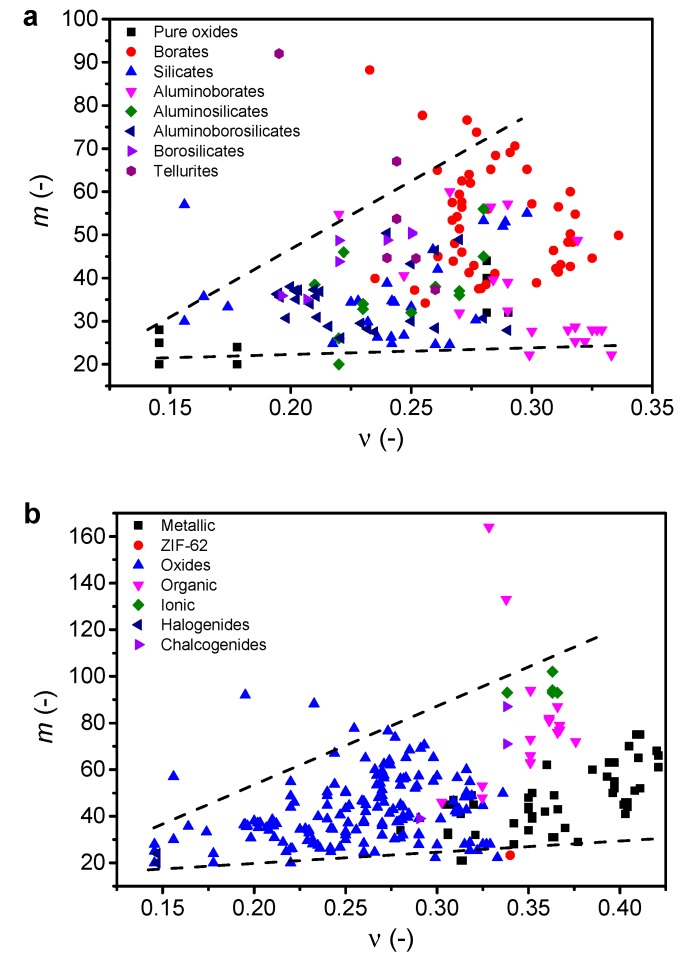
Liquid fragility (*m*) as function of Poisson’s ratio (*ν*) for (**a**) oxide glass-formers (including both present compositions from Table 1 and literature data) and (**b**) various glass-formers from literature. References for literature data are given in the text. The dashed lines are guides for the eye, showing the trends for the majority of the data. The errors associated with *m* and *ν* are 1 and 0.01, respectively. *R*^2^ values for linear fits to the data are 0.078 and 0.178 for (**a**) and (**b**), respectively.

Next, we revisit the correlation between liquid fragility and Poisson’s ratio. Considering first the oxide glasses from Table 1 with *ν* ≥ 0.28, we find no apparent correlation between *ν* and *m* (Figure 4). Hence, the data for these oxide glass-formers with relatively high *ν* values support the criticism of the Novikov and Sokolov correlation [8,41]. Figure 5 further tests the *m*-*ν* correlation by including literature data on various oxide glass formers (Figure 5a) and all types of glass families (Figure 5b). The oxide glass-formers include pure oxides (SiO_2_, B_2_O_3_, GeO_2_) [30,151], borates [18,64,67,76,78,151,152,153], silicates [18,89,152,154], aluminoborates [18], aluminoborosilicates [18,101], borosilicates [18,101], and tellurites [114,121,155]. For these systems, we highlight two observations. First, multiple liquid fragility data are obtained for pure oxides though having same (or very similar) Poisson’s ratio, e.g., three data points are shown in Figure 5a for SiO_2_ (*ν* = 0.145 [31]). Second, for some glass systems the values of *m* and *ν* are obtained from different studies when only one of the properties is reported. In those cases, we have compared the density, molar volume, or atomic packing density values to ensure the similarity of the materials. The additional glasses in Figure 5b include metallic [38,39,40,156], ZIF-62 [157], organic [30,31], ionic [30,151], halogenide [30], and chalcogenide glasses [30]. 

There appears to be a weak positive correlation between liquid fragility and Poisson’s ratio, but it is significantly scattered and not universal as previously reported for organic, inorganic, and metallic glasses [41]. Here, we have also included oxide glasses, but these do not strengthen the possible correlation between *m* and *ν*. We note that Greaves et al. [6] have shown a positive *m*-*ν* correlation with varying slope for each glass system. The oxide glass systems used in that study were binary silicates, but as seen in Figure 5a, there is no strong correlation when considering a wider range of oxide glass families. It thus appears that the correlation can only be found within very narrow compositional variations, as those in binary sodium or potassium silicates. When considering all the glass families (Figure 5b), it is also evident that no universal correlation is observed, since a very wide range of *ν* values (around 0.15 to 0.40) is seen for a relatively narrow range of *m* values (around 25 to 35). 

### 3.4. Implications for Design of Tough Oxide Glasses

In order to design mechanically tough oxide glasses, it is important to control the factors that influence the fracture toughness (*K*_Ic_). Therefore, besides fracture energy (*G*_frac_) and Poisson’s ratio (Figure 1), we analyze the effect of Young’s modulus (*E*) on *K*_Ic_. Under plane strain, we have [158],

(6) $$K_{Ic}=\sqrt{\frac{G_{frac}\;E}{\left(1-\nu^{2}\right)}}$$

**Figure 6:**
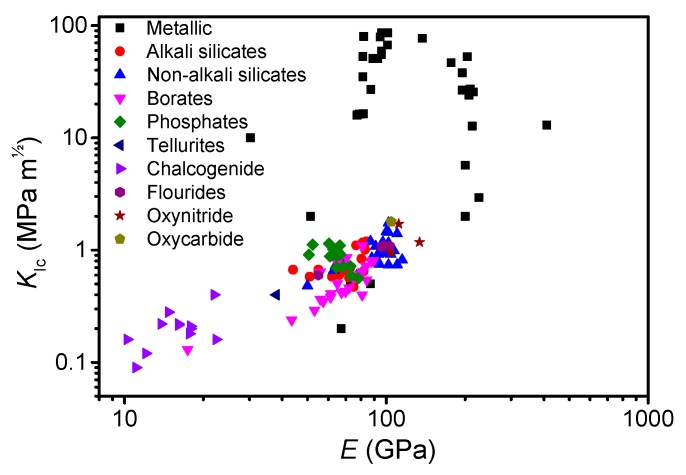
Dependence of measured fracture toughness (*K*_Ic_) on the measured Young’s modulus (*E*) for various glass systems (references are given in the text). Note that the axes are logarithmic. Errors in *K*_Ic_ and *E* are estimated to be smaller than 0.05 MPa m^½^ and 2 GPa, respectively.

To understand what criteria need to be fulfilled to design high-*K*_Ic_ oxide glasses, we have calculated *G*_frac_ for various systems based on the measured literature values of *K*_Ic_, *E*, and *ν*. Glasses from literature include metallic [9,20,159,160,161,162,163], silicates [18,19,20,21,82,164,165,166], borates [20,166,167], phosphates [126,166], tellurites [166], chalcogenides [20,166,168,169], flourides and oxyflourides [164,166], oxynitrides [20], and oxycarbides [20]. As discussed, there is a pronounced effect of *ν* on *G*_frac_ when *ν* exceeds ~0.32 (Figure 1). In contrast, there is no correlation between *E* and *ν* (see Figure S2 in the Supporting Information), whereas there is an expected correlation between *K*_Ic_ and *G*_frac_ (see Figure S3 in the Supporting Information). Even though only *ν* affects *G*_frac_, *K*_Ic_ is also increasing with *E* (Figure 6). This highlights the importance of tailoring future glass composition with a combination of high *G*_frac_ and *E*. In turn, this confirms the importance of producing high-*ν* oxide glasses (due to the *ν-G*_frac_ relation) in order to improve the fracture toughness. We note that the correlation between *K*_Ic_ and *E* for metallic glasses is vague (Figure 6), which might be due to the difficulty in measuring *K*_Ic_ of metallic glasses [170,171], while *ν*, in contrast, is easy to measure. In summary, there are three ways to increase the fracture energy: (i) increase the ductility (or by surrogate Poisson’s ratio), (ii) increase the ultimate strain at constant *E*, and/or (iii) increase *E* at constant ultimate strain. All of these increase the area under the stress-strain curve, but note that (ii) and (iii) assume that fracture remains fully brittle.

## 4. Conclusions

We have tested the validity of previously proposed relationships between Poisson’s ratio on the one hand and liquid fragility and atomic packing density on the other hand. This was done by performing an extensive literature review and by preparing new oxide glasses, especially within the zinc borate and aluminoborate families that exhibit Poisson’s ratio (*ν*) above 0.30, up to 0.34. This is relevant for oxide glasses, since these are believed to undergo a brittle-to-ductile transition for *ν*~0.32. Although two overall increasing trends in Poisson’s ratio with both liquid fragility and atomic packing density are observed, it is also clear that no universal relationships are observed when considering the wide range of compositions herein, including oxide, metallic, halogenide, chalcogenide, ionic, and organic glass families. This work suggests that additional structural details besides, e.g., packing density, are needed to predict the Poisson’s ratio of oxide glasses. 

## Figures and Tables

**Table 1:** Atomic packing factor (*C*_g_), glass transition temperature (*T*_g_), liquid fragility (*m*), and Poisson’s ratio (*ν*) of various oxide glasses, either synthesized for this work or taken from previous studies [18,46,47,48,49,52]. The errors in *C*_g_, *T*_g_, *m*, and *ν* are estimated to be within ±0.002, 2 °C, 1, and 0.01, respectively.

Composition (mol%)		*C*_g_ (-)	*T*_g_ (°C)	*m* (-)	*ν* (-)
Ca-Zr-Silicates	45CaO-5ZrO_2_-50SiO_2_	0.523	789.2	53.3	0.280
	50CaO-5ZrO_2_-45SiO_2_	0.524	806.2	52.0	0.288
Zn-Borates	55ZnO-45B_2_O_3_^(a)^	0.566 ^(a)^	556.5	57.2	0.300 ^(a)^
	2La_2_O_3_-53ZnO-45B_2_O_3_^(a)^	0.565 ^(a)^	557.4	56.5	0.311 ^(a)^
	5La_2_O_3_-50ZnO-45B_2_O_3_^(a)^	0.572 ^(a)^	565.3	60.0	0.316 ^(a)^
	10La_2_O_3_-45ZnO-45B_2_O_3_^(a)^	0.580 ^(a)^	552.4	54.8	0.318 ^(a)^
	5La_2_O_3_-10GeO_2_-50ZnO-35B_2_O_3_	0.554	576.3	41.4	0.311
	2Ta_2_O_5_-53ZnO-45B_2_O_3_	0.581	559.6	42.7	0.316
	5Ta_2_O_5_-50ZnO-45B_2_O_3_	0.577	563.7	48.3	0.315
	2Ta_2_O_5_-55ZnO-43B_2_O_3_	0.583	547.6	49.9	0.336
	5Ta_2_O_5_-55ZnO-40B_2_O_3_	0.550	563.7	48.3	0.317
	10Sb_2_O_3_-55ZnO-35B_2_O_3_	0.498	502.4	39.9	0.278
	2La_2_O_3_-55ZnO-43B_2_O_3_	0.583	533.0	38.9	0.302
	5La_2_O_3_-55ZnO-40B_2_O_3_	0.551	557.1	46.4	0.309
	10La_2_O_3_-55ZnO-35B_2_O_3_	0.539	542.2	42.2	0.310
	2La_2_O_3_-2Ta_2_O_5_-53ZnO-43B_2_O_3_	0.617	538.7	50.2	0.316
	5La_2_O_3_-2Ta_2_O_5_-50ZnO-43B_2_O_3_	0.580	539.5	44.6	0.325
	5La_2_O_3_-5Ta_2_O_5_-50ZnO-40B_2_O_3_	0.569	547.2	43.1	0.312
Aluminoborates	25MgO-20Al_2_O_3_-55B_2_O_3_^(b)^	0.565 ^(b)^	636 ^(b)^	56.5	0.283
	25CaO-20Al_2_O_3_-55B_2_O_3_^(b)^	0.551 ^(b)^	615 ^(b)^	54.8	0.220
	25SrO-20Al_2_O_3_-55B_2_O_3_^(b)^	0.537 ^(b)^	590 ^(b)^	60.0	0.266
	25BaO-20Al_2_O_3_-55B_2_O_3_^(b)^	0.545 ^(b)^	554 ^(b)^	57.2	0.290
	18.75Li_2_O-6.25BaO-20Al_2_O_3_-55B_2_O_3_	0.531	484.4	39.6	0.284
	20Li_2_O-5MgO-20Al_2_O_3_-55B_2_O_3_^(c)^	0.553 ^(c)^	482 ^(c)^	40.6	0.247
	25Cs_2_O-20Al_2_O_3_-55B_2_O_3_^(d)^	0.479 ^(d)^	416 ^(d)^	48.8	0.319 ^(d)^
	25Cs_2_O-5Ga_2_O_3_-15Al_2_O_3_-55B_2_O_3_	0.480	421.2	28.0	0.324
	25Cs_2_O-10Ga_2_O_3_-10Al_2_O_3_-55B_2_O_3_	0.474	418.7	25.3	0.320
	25Cs_2_O-2Ta_2_O_3_-18Al_2_O_3_-55B_2_O_3_	0.474	432.1	28.7	0.318
	23Cs_2_O-2Ta_2_O_3_-20Al_2_O_3_-55B_2_O_3_	0.470	433.6	22.2	0.316
	21Cs_2_O-4Ta_2_O_3_-20Al_2_O_3_-55B_2_O_3_	0.475	449.7	27.7	0.300
Aluminoborosilicates	25Na_2_O-75SiO_2_^(e)^	0.49 ^(e)^	475 ^(e)^	33.3	0.25 ^(e)^
	25Na_2_O-12.5B_2_O_3_-62.5SiO_2_^(e)^	0.52 ^(e)^	539 ^(e)^	43.8	0.22 ^(e)^
	25Na_2_O-25B_2_O_3_-50SiO_2_^(e)^	0.55 ^(e)^	544 ^(e)^	48.7	0.22 ^(e)^
	25Na_2_O-37.5B_2_O_3_-37.5SiO_2_^(e)^	0.56 ^(e)^	525 ^(e)^	48.8	0.24 ^(e)^
	25Na_2_O-50B_2_O_3_-25SiO_2_^(e)^	0.56 ^(e)^	511 ^(e)^	50.6	0.25 ^(e)^
	25Na_2_O-62.5B_2_O_3_-12.5SiO_2_^(e)^	0.56 ^(e)^	495 ^(e)^	50.2	0.25 ^(e)^
	25Na_2_O-75B_2_O_3_^(e)^	0.56 ^(e)^	473 ^(e)^	51.4	0.27 ^(e)^
	25Na_2_O-12.5Al_2_O_3_-62.5SiO_2_^(e)^	0.49 ^(e)^	567 ^(e)^	32.8	0.23 ^(e)^
	25Na_2_O-12.5Al_2_O_3_-12.5B_2_O_3_-50SiO_2_^(e)^	0.51 ^(e)^	545 ^(e)^	50.4	0.24 ^(e)^
	25Na_2_O-12.5Al_2_O_3_-25B_2_O_3_-37.5SiO_2_^(e)^	0.52 ^(e)^	514 ^(e)^	43.3	0.25 ^(e)^
	25Na_2_O-12.5Al_2_O_3_-37.5B_2_O_3_-25SiO_2_^(e)^	0.52 ^(e)^	493 ^(e)^	46.4	0.26 ^(e)^
	25Na_2_O-12.5Al_2_O_3_-50B_2_O_3_-12.5SiO_2_^(e)^	0.52 ^(e)^	480 ^(e)^	48.9	0.27 ^(e)^
	25Na_2_O-12.5Al_2_O_3_-62.5B_2_O_3_^(e)^	0.52 ^(e)^	465 ^(e)^	39.0	0.29 ^(e)^
	25Na_2_O-25Al_2_O_3_-50SiO_2_^f)^	0.49 ^(e)^	792 ^(e)^	38.5	0.21 ^(e)^
	25Na_2_O-25Al_2_O_3_-12.5B_2_O_3_-37.5SiO_2_^(e)^	0.49 ^(e)^	611 ^(e)^	30.0	0.25 ^(e)^
	25Na_2_O-25Al_2_O_3_-25B_2_O_3_-25SiO_2_^(e)^	0.49 ^(e)^	511 ^(e)^	28.4	0.26 ^(e)^
	25Na_2_O-25Al_2_O_3_-37.5B_2_O_3_-12.5SiO_2_^(e)^	0.50 ^(e)^	468 ^(e)^	30.7	0.28 ^(e)^
	25Na_2_O-25Al_2_O_3_-50B_2_O_3_^(e)^	0.50 ^(e)^	459 ^(e)^	32.4	0.29 ^(e)^
	25Na_2_O-30Al_2_O_3_-45B_2_O_3_^(e)^	0.50 ^(e)^	528 ^(e)^	31,9	0.27 ^(e)^
	25Na_2_O-30Al_2_O_3_-32.5B_2_O_3_-12.5SiO_2_^(e)^	0.50 ^(e)^	469 ^(e)^	27.9	0.29 ^(e)^

^(a)^ Glasses and/or data are from Ref. [52]; ^(b)^ Glasses and/or data are from Ref. [48]; ^(c)^ Glasses and/or data are from Ref. [46]; ^(d)^ Glasses and/or data are from Ref. [18]; ^(e)^ Glass is from Ref. [49].

## Supplementary Materials

The following are available online at https://www.mdpi.com/1996-1944/12/15/2439/s1, Calculation of atomic packing density (*C*_g_) and the structural assumptions made. **Figure S1:** Dependence of Poisson’s ratio (*ν*) on atomic packing density (*C*_g_) for various glass systems, including those from Table 1. References for literature data are given in the main text. *C*_g_ is calculated according to Equation (3), building on the structural assumptions described in the Supporting Information. The errors associated with *ν* and *C*_g_ are smaller than the size of the symbols (0.01 and 0.002, respectively). The empirical Makishima-Mackenzie model (MM-model, solid line) [43] is also represented (black line). **Figure S2.** Dependence of Young’s modulus (*E*) on Poisson’s ratio (*ν*) for various glass systems. References for all data are given in the main text. Errors on *E* and *ν* are estimated to be smaller than 2 GPa and 0.01, respectively. **Figure S3.** Dependence of measured fracture toughness (*K*_Ic_) on the calculated fracture energy (*G*_frac_) for various glass systems (references are given in the main text). Note that the axes are logarithmic and that *K*_Ic_ (together with *E* and *ν*) are used in the calculation of *G*_frac_ based on Equation (6) from the main manuscript. Errors in *K*_Ic_ and *G*_frac_ are estimated to be smaller than 0.05 and 15%, respectively.
