# Supplementary material for: Revisiting the Dependence of Poisson’s Ratio on Liquid Fragility and Atomic Packing Density in Oxide Glasses
Source: Materials (Basel). 2019 Jul 31;12(15):2439. doi: 10.3390/ma12152439 (PMC6696245; doi:10.3390/ma12152439)
Supplement: Supplementary file 1 [file materials-12-02439-s001.pdf]

materials

MDPI

Supporting Information

Revisiting the Dependence of Poisson's Ratio on Liquid Fragility and Atomic Packing Density in Oxide Glasses

Martin B. Østergaard [1], Søren R. Hansen [1], Kacper Januchta [1], Theany To [1], Sylwester J. Rzoska [2], Michal Bockowski [2], Mathieu Bauchy [3] and Morten M. Smedskjaer [1,*]

[1] Department of Chemistry and Bioscience, Aalborg University, 9220 Aalborg East, Denmark
[2] Institute of High-Pressure Physics, Polish Academy of Sciences, Warsaw 01-142, Poland
[3] Department of Civil and Environmental Engineering, University of California, Los Angeles, CA 90095, USA
*Corresponding author. E-mail: mos@bio.aau.dk

Calculation of atomic packing density (C_g)

In addition to density and chemical composition, the atomic-scale structure of glasses greatly affects the calculation of the atomic packing density (C_g). Many studies do not report the required structural features, including coordination number and valence state. We have thus made a number of assumptions for the calculation of C_g, since C_g is only rarely reported. To calculate C_g, we have used the ionic radii of the species reported by Shannon [1] and the following structural assumptions, in addition to the reported chemical composition and density (molar volume).

General assumptions: In all glass systems, alkali, alkaline-earth, and rare-earth modifiers are assumed to be in six-fold coordination, unless anything else is stated in the references. We note that this is not always the case, but made this assumption for reasons of simplicity and lacking structural analyses. However, for some glass systems we have changed the coordination, e.g., to eight-fold, when described in the literature. Another common assumption is that silicon is always in four-fold coordination. Therefore, all alkali silicate, alkaline earth silicate, and alkali-alkaline earth silicate glasses are assumed to contain six-fold coordinated modifiers and four-fold coordinated silicon species.

Borates: In alkali borate glasses, boron changes coordination state from three-fold to four-fold with increasing content of modifier. The fraction of four-fold boron (N_4) is here calculated as $N_4 = x/(1-x)$, where x is the molar modifier content [2]. For alkali alkaline earth borates, we used the sum of alkali and alkaline earth modifiers to calculate x and thus N_4. Zinc borate glasses have been synthesized for this study and reported in literature [3,4]. Zinc typically exists in four- or six-fold coordination [5], however, in these glass systems, we assume pure four-fold zinc due to its high concentration and boron in three-fold coordination [6,7]. However, Gaafar *et al.* [3] reports N_4 values up to 35% for a sodium zing aluminoborate system. The boron coordination does not significantly affect the calculation of C_g calculations due to the small difference in ionic radii of trigonal and tetrahedral boron. Additionally, the samples prepared by Gaafar *et al.* [3] contain small amounts of alumina that are assumed to be in four-fold coordination due to the small alumina content (< 4 mol%) and reported structural data on aluminoborates, showing 97% of aluminum in four-fold coordination [8]. Lanthanum occur in various coordination states, but the most common seems to be eight-fold [9], which is thus used for all compositions. The difference in the value of C_g for lanthanum containing zinc borates is less than ± 2% when changing the coordination number of zinc and lanthanum from six- to four-fold and six- to eight-fold, respectively. For lead borate glasses, we face the same problem with four- and six-fold coordinated lead as for zinc in zinc borates. However, lead is mainly found in six-fold coordination, since lead borates with 50% PbO has an average coordination number of 5.1 [10]. In manganese borates, manganese is assumed to be divalent in six-fold coordination [7].

Aluminoborates and -silicates: Aluminoborate glasses are both synthesized in this study and reported in literature [8,11–13]. In these glasses, we use the structural date reported by Januchta *et al.*

[8] to calculate C_g. They report 92% three-fold and 8% four-fold boron, and 97% four-fold, 2% five-fold, and 1% six-fold aluminum. Additionally, we synthesized aluminoborates with alumina substituted by gallia. Gallium is mainly in tetrahedral configuration [14], and we therefore assume gallium to act as aluminum in the glasses [15], and thus, use the same distribution of four-, five-, and six-fold coordination. In the studied aluminosilicate glasses, aluminum is mainly in four-fold coordination (> 99%) [16], and thus, we assume pure four-fold coordination.

Germanates: Germanium is mainly in four- and/or six-fold coordination. Though, the coordination number of germanium is not as easy to predict as for, e.g., boron and in literature multiple suggestions are found. For simplicity, we have followed the suggestion by Osaka et al. [17] describing the amount of six-fold coordinated germanium to equal the amount of modifier added to the glass system, and thus, the remaining part is four-fold. We use this assumption for both germanate and germanosilicate glasses.

Phosphates: We generally assume that phosphorus is in four-fold coordination. We use data for titanophosphate glasses containing alumina [18], for which we assume 80% six-fold and 20% four-fold titanium as Ti^{4+} is mainly found in octahedral coordination [19]. In addition, aluminum is found to be in six-fold coordination [19]. In other cases, titanium is shown to be mainly five-fold (60%), while it is 25% four-fold and 15% six-fold coordinated [20,21]. We use this distribution of species in silicate glasses with up to 20 mol% TiO_2 [12,22,23]. Cobalt phosphate glasses [24] contain a combination of CoO and Co_2O_3, hence, divalent and trivalent cobalt ions. All Co^{3+} is six-fold coordinated, while Co^{2+} is in a combination of four-fold and six-fold coordination [25]. In the binary system, the difference is < 2.5% when the CoO+Co_2O_3 content is < 20 mol%. At higher CoO+Co_2O_3 contents, the difference in coordination state increases due to the incorporation of more oxygen for Co_2O_3 compared to CoO, with the difference reaching 11% at 60 mol% CoO + Co_2O_3. Therefore, we assume an equal distribution of CoO and Co_2O_3 in the phosphate glasses [19].

We have also included literature data for phosphate glasses containing large amounts of tungsten oxide or molybdenum oxide [26–28]. As for other phosphates, phosphorus is assumed to be four-fold. The structural units of tungsten and molybdenum oxides are reported as hexavalent [26–28], but they can also occur as pentavalent [29,30]. The higher the melting temperature, the higher fraction of pentavalent ions is observed [30]. For the calculation of packing density, the coordination numbers of tungsten and molybdenum can potentially be 4 to 6 and 4 to 7, respectively. Tungsten and molybdenum are mainly octahedrally coordinated, while high alkali oxide content causes tetrahedral units [26]. Therefore, taking into account the high melting temperatures utilized (mainly >1200 K), we assume a mixture of 80% penta- and 20% hexavalent tungsten and molybdenum in all glasses. The coordination is assumed to be pure six-fold tungsten and molybdenum in binary phosphate glasses, while an equal mix of four- and six-fold in ternary tungsten or molybdenum phosphates is assumed. The change in valence state has a significant influence on C_g, while the coordination number has only a smaller effect (< 1.5%) on C_g for a high-molybdenum phosphate ($80MoO_3$-$20P_2O_5$). Another reason for assuming some pentavalent tungsten is that a $80WO_3$-$20P_2O_5$ glass would otherwise exhibit an unrealistic high value of C_g (0.974). With the mentioned assumptions, the C_g of that composition is 0.871, which is high, yet more realistic.

Copper phosphate glasses with different copper valence state are characterized in literature [31]. The ratio of Cu^+ and Cu^{2+} is reported, and thus, it is used to calculate the C_g. Cu^+ and Cu^{2+} act differently, as Cu^+ shows a covalent bonding nature and Cu^{2+} is ionic acting as modifier [32,33]. Therefore, we use Cu^+ as two-fold and Cu^{2+} as four-fold in our calculations [33].

Tellurites: Tellurite glasses are reported with various compositions. Tellurium exists in four-fold coordination in glasses without modifier, but with increasing modifier content, the coordination number changes towards three-fold with 50% N_3 and 50% N_4 for a $80TeO_2$-$20K_2O$ glass [34]. We here calculate the fraction of three-fold coordinated tellurium as $N_3(\%) = 200y/(100-y)$, where y is the amount of alkali oxide. The remaining tellurium is assumed to be four-fold [34]. Other tellurite glasses contain vanadium or bismuth that are five-fold and three- or six-fold coordinated, respectively [35,36]. We assume purely six-fold bismuth in glasses as there are no radii data on three-fold coordinated bismuth from Shannon [1], while vanadium is assumed to be five-fold. Cerium and yttrium are reported as eight- and seven-fold coordinated, respectively [37].

Vanadates: Vanadate glasses are typically build up by V^{5+} based networks. However, for compositions with 40–50 mol% of MO (M=divalent cation such as Ca^{2+}, Pb^{2+} etc.), the main oxidation state of vanadium is V^{4+} [38]. The vanadate glasses considered from literature [39,40] contain 50 to 80 mol% V_2O_5, and therefore, we assume an equal mix of V^{4+} and V^{5+} in all glasses, both as five-fold coordinated.

Dependence of Poisson's ratio on atomic packing density for all studied glasses

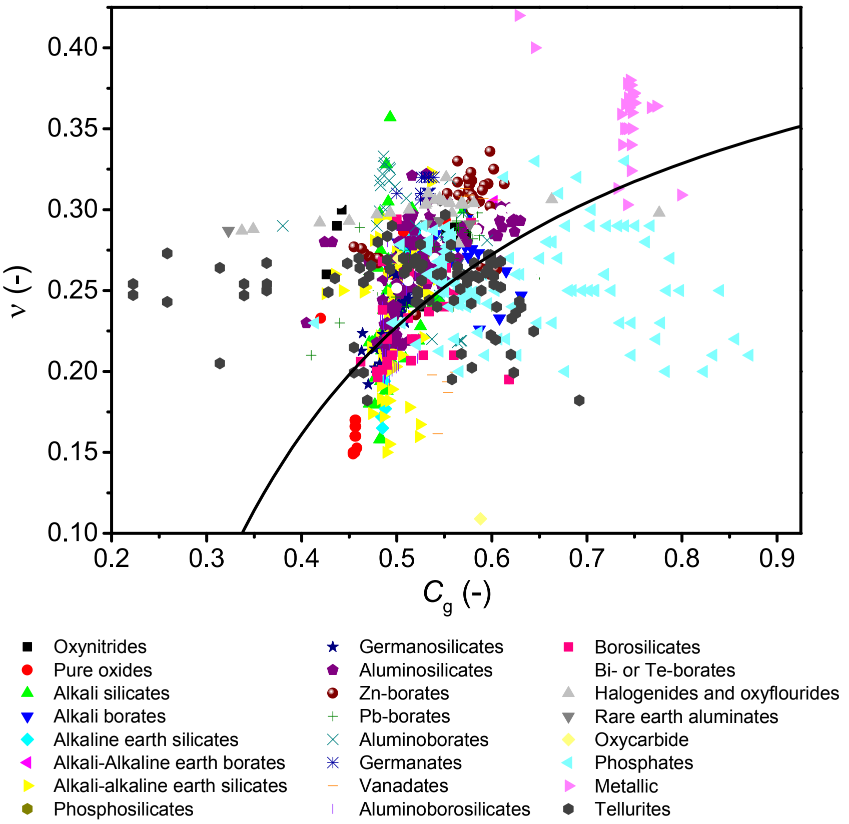

Figure S1. Dependence of Poisson's ratio (ν) on atomic packing density (C_g) for various glass systems, including those from Table 1. References for literature data are given in the main text. C_g is calculated according to Eq. (3), building on the structural assumptions described in the Supporting Information. The errors associated with ν and C_g are smaller than the size of the symbols (0.01 and 0.002, respectively). The empirical Makishima-Mackenzie model (MM-model, solid line) [41] is also represented (black line).

Correlation between Young's modulus and Poisson's ratio

The dependence of Young's modulus (E) on Poisson's ratio (ν) for various glass systems is investigated. We note that within a relatively narrow range in E (70-100 GPa), a large range of ν values (0.15-0.42) is possible.

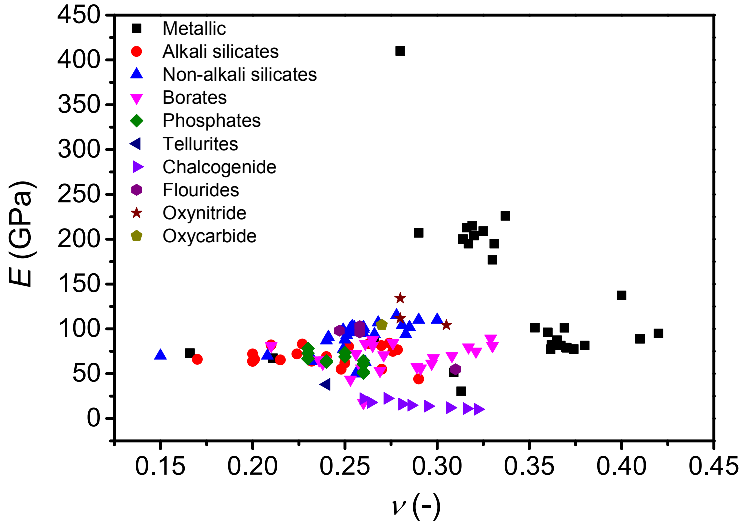

Figure S2. Dependence of Young's modulus (E) on Poisson's ratio (ν) for various glass systems. References for all data are given in the main text. Errors on E and ν are estimated to be smaller than 2 GPa and 0.01, respectively.

Correlation between fracture toughness and fracture energy

The fracture toughness (K_{Ic}) depends on the fracture energy (G_{frac}) in the calculation (Eq. 6 in main manuscript) under plain strain. Based on K_{Ic} values and corresponding Young's modulus (E) and Poisson's ratio (ν) values from literature, we have calculated G_{frac}. As shown in Figure S3, there is a linear correlation in a log-log plot of K_{Ic} vs. G_{frac}. The correlation is expected since G_{frac} is partly calculated based on K_{Ic} (but also E and ν). Therefore, it is of interest to control G_{frac}, which is possible by obtaining materials with high Poisson's ratio.

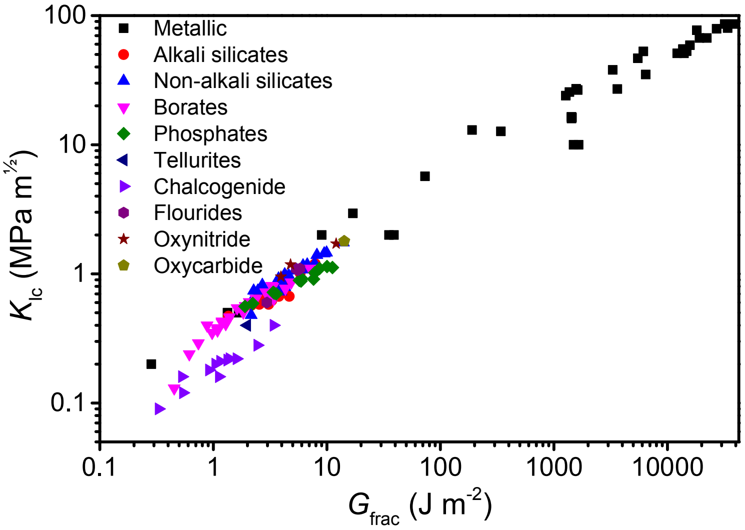

Figure S3. Dependence of measured fracture toughness (K_{Ic}) on the calculated fracture energy (G_{frac}) for various glass systems (references are given in the main text). Note that the axes are logarithmic and that K_{Ic} (together with E and ν) are used in the calculation of G_{frac} based on Eq. 6 from the main manuscript. Errors in K_{Ic} and G_{frac} are estimated to be smaller than 0.05 and 15%, respectively.

Supporting References

1. R.D. Shannon, Revised effective ionic radii and systematic studies of interatomic distances in halides and chaleogenides, Acta Crystallogr. A32 (1976) 751–767.

2. J. Zhong, P.J. Bray, Change in boron coordination in alkali borate glasses, and mixed alkali effects, as elucidated by NMR, J. Non. Cryst. Solids. 111 (1989) 67–76.
3. M.S. Gaafar, N.S.A. El-aal, O.W. Gerges, G. El-amir, Elastic properties and structural studies on some zinc-borate glasses derived from ultrasonic , FT-IR and X-ray techniques, J. Alloys Compd. 475 (2009) 535–542.
4. A. Kannappan, S. Thirumaran, R. Palani, Elastic and mechanical properties of glass specimen by ultrasonic method, J. Eng. Appl. Sci. 4 (2009) 27–31.
5. D. Ehrt, Zinc and manganese borate glasses – phase separation , crystallisation , photoluminescence and structure, Phys. Chem. Glas. Eur. J. Glas. Sci. Technol. B. 54 (2013) 65–75.
6. A. Kajinami, Y. Harada, S. Inoue, S. Deki, N. Umesaki, The structural analysis of zinc borate glass by laboratory EXAFS and X-ray diffraction measurements, Jpn. J. Appl. Phys. 38 (1999) 132–135.
7. D. Möncke, E.I. Kamitsos, D. Palles, R. Limbach, A. Winterstein-Beckmann, T. Honma, Z. Yao, T. Rouxel, L. Wondraczek, Transition and post-transition metal ions in borate glasses : Borate ligand speciation , cluster formation , and their effect on glass transition and mechanical properties, J. Chem. Phys. 145 (2016) 124501.
8. K. Januchta, M. Bauchy, R.E. Youngman, S.J. Rzoska, M. Bockowski, M.M. Smedskjaer, Modifier field strength effects on densification behavior and mechanical properties of alkali aluminoborate glasses, Phys. Rev. Mater. 1 (2017) 063603.
9. C.W. Ponader, G.E. Brown, Rare earth elements in silicate glass/melt systems: I. Effects of composition on the coordination environments of La , Gd , and Yb, Geochim. Cosmochim. Acta. 53 (1989) 2893–2903.
10. T. Takaishi, J. Jin, T. Uchino, T. Yoko, Structural Study of PbO–B_2O_3 Glasses by X-ray Diffraction and ^{11}B MAS NMR Techniques, J. Am. Ceram. Soc. 48 (2000) 2543–2548.
11. K. Januchta, T. To, M.S. Bødker, T. Rouxel, M.M. Smedskjaer, Elasticity, hardness, and fracture toughness of sodium aluminoborosilicate glasses, J. Am. Ceram. Soc. (2019) 1–18.
12. A.A. El-moneim, Interpretation of elastic properties and structure of TiO2–CaO–Al2O3–B2O3glasses, Phys. Chem. Glas. 45 (2004) 15–20.
13. K. Januchta, R. Sun, L. Huang, M. Bockowski, S.J. Rzoska, L.R. Jensen, M.M. Smedskjaer, Deformation and cracking behavior of La_2O_3-doped oxide glasses with high Poisson ' s ratio, J. Non. Cryst. Solids. 494 (2018) 86–93.
14. F. Miyaji, S. Sakka, Structure of PbO-Bi_20_3-Ga_20_3 glasses, J. Non. Cryst. Solids. 134 (1991) 77–85.
15. J.L. Piguet, J.E. Shelby, Transformation-Range Behavior of Li2O-(Al,Ga)2O3-SiO2 Glasses, Commun. Am. Ceram. Soc. 68 (1985) 232–233.
16. T.K. Bechgaard, A. Goel, R.E. Youngman, J.C. Mauro, S.J. Rzoska, M. Bockowski, L.R. Jensen, M.M. Smedskjaer, Structure and mechanical properties of compressed sodium aluminosilicate glasses: Role of non-bridging oxygens, J. Non. Cryst. Solids. 441 (2016) 49–57.
17. A. Osaka, K. Ariyoshi, K. Takahashi, Network structure of alkali germanosilicate glasses, J. Non. Cryst. Solids. 83 (1986) 335–343.
18. M.N. Svenson, M. Guerette, L. Huang, N. Lönnroth, J.C. Mauro, S.J. Rzoska, M. Bockowski, M.M. Smedskjaer, Universal behavior of changes in elastic moduli of hot compressed oxide glasses, Chem. Phys. Lett. 651 (2016) 88–91.
19. L.A. Farrow, E.M. Vogel, Raman spectra of phosphate and silicate glasses doped with the cations Ti , Nb and Bi, J. Non. Cryst. Solids. 143 (1992) 59–64.
20. F. Farges, Ab initio and experimental pre-edge investigations of the Mn K-edge XANES in oxide-type materials, Phys. Rev. B - Condens. Matter Mater. Phys. 71 (2005) 1–14.
21. M. Schneider, W. Richter, R. Keding, C. Rüssel, XPS investigations on coordination and valency of Ti in fresnoite glasses and glass ceramics, J. Non. Cryst. Solids. 226 (1998) 273–280.
22. A. Makishima, Y. Tamura, T. Sakaino, Elastic Moduli and Refractive Indices of Aluminosilicate Glasses Containing Y2O3, La2O3, and Ti02, J. Am. Ceram. Soc. 61 (1978) 247–249.
23. G. Scannell, D. Laille, L. Huang, T. Rouxel, Interaction between deformation and crack initiation under Vickers indentation in Na_2O-TiO_2-SiO_2 glasses, Front. Mater. 4 (2017) 1–10.
24. A.A. Higazy, B. Bridge, Elastic constants and structure of the vitreous system Co_3O_4-P_2O_5, J. Non. Cryst. Solids. 72 (1985) 81–108.
25. T. Maekawa, T. Yokokawa, K. Niwa, Optical Spectra of Transition in Na_2O-P_2O_5 Glasses, Bull. Chem. Soc. Jpn. 42 (1969) 2102–2106.
26. S. Muthupari, S. Lakshmi Raghavan, K.J. Rao, Elastic properties of binary AO3-P2O5 and ternary Na2O-AO3-P2O5 (A ≡ Mo or W) glasses, Mater. Sci. Eng. B. 38 (1996) 237–244.

27. K. V Damodaran, K.J. Rao, Elastic Properties of Alkali Phosphomolybdate Glasses, J. Am. Ceram. Soc. 39 (1989) 5–11.
28. K. V Damodaran, K.J. Rao, Elastic properties of phosphotungstate glasses, J. Mater. Sci. 24 (1989) 2380–2386.
29. D. Boudlich, L. Bih, M.E.H. Archidi, M. Haddad, A. Yacoubi, A. Nadiri, B. Elouadi, Infrared, Raman, and Electron Spin Resonance Studies of Vitreous Alkaline Tungsten Phosphates and Related Glasses, J. Am. Ceram. Soc. 85 (2010) 623–630.
30. G. Poirier, F.S. Ottoboni, F.C. Cassanjes, Á. Remonte, Y. Messaddeq, S.J.L. Ribeiro, Redox behavior of molybdenum and tungsten in phosphate glasses, J. Phys. Chem. B. 112 (2008) 4481–4487.
31. T. Miura, T. Watanabe, Y. Benino, T. Komatsu, Unusual Elastic and Mechanical Behaviors of Copper Phosphate Glasses with Different Copper Valence States, J. Am. Ceram. Soc. 84 (2001) 2401–2408.
32. R. Sato, T. Komatsu, K. Matusita, Unique physical properties and fragility of 50CuOx-50P205 glasses, J. Non. Cryst. Solids. 201 (1996) 222–230.
33. K. Kamiya, K. Okasaka, M. Wada, H. Nasu, T. Yoko, Extended x-ray absorption fine structure (EXAFS) study on the local environment around copper in low thermal expansion copper aluminosilicate glasses, J. Am. Ceram. Soc. 75 (1992) 477–478.
34. M.A. Sidkey, M.S. Gaafar, Ultrasonic studies on network structure of ternary TeO_2–WO_3–K_2O glass system, Phys. B. 348 (2004) 46–55.
35. N. Elkhoshkhany, R. El-Mallawany, E. Syala, Mechanical and thermal properties of TeO_2-Bi_2O_3-V_2O_5-Na_2O-TiO_2 glass system, Ceram. Int. 42 (2016) 19218–19224.
36. V. Dimitrov, Y. Dimitriev, A. Montenero, IR spectra and structure of V_2O_5-GeO_2-Bi_20_3 glasses, J. Non. Cryst. Solids. 180 (1994) 51–57.
37. R. El-Mallawany, Quantitative analysis of elastic moduli of tellurite glasses, J. Mater. Res. 5 (1990) 2218–2222.
38. Y. Dimitriev, V. Dimitrov, M. Arnaudov, D. Topalov, IR-spectral study of vanadate vitreous systems, J. Non. Cryst. Solids. 57 (1983) 147–156.
39. Y.B. Saddeek, E.R. Shaaban, K.A. Aly, I.M. Sayed, Characterization of some lead vanadate glasses, J. Alloys Compd. 478 (2009) 447–452.
40. V. Rajendran, N. Palanivelu, D.K. Modak, B.K. Chaudhuri, Ultrasonic investigation on ferroelectric BaTiO3 doped 80V2O5-20PbO oxide glasses, Phys. Status Solidi. 180 (2000) 467.
41. A. Makishima, J.D. Mackenzie, Calculation of bulk modulus, shear modulus, and Poisson's ratio of glass, J. Non. Cryst. Solids. 17 (1975) 147–157.
